# A Mutation in the Mitochondrial Fission Gene *Dnm1l* Leads to Cardiomyopathy

**DOI:** 10.1371/journal.pgen.1001000

**Published:** 2010-06-24

**Authors:** Houman Ashrafian, Louise Docherty, Vincenzo Leo, Christopher Towlson, Monica Neilan, Violetta Steeples, Craig A. Lygate, Tertius Hough, Stuart Townsend, Debbie Williams, Sara Wells, Dominic Norris, Sarah Glyn-Jones, John Land, Ivana Barbaric, Zuzanne Lalanne, Paul Denny, Dorota Szumska, Shoumo Bhattacharya, Julian L. Griffin, Iain Hargreaves, Narcis Fernandez-Fuentes, Michael Cheeseman, Hugh Watkins, T. Neil Dear

**Affiliations:** 1 Department of Cardiovascular Medicine and Wellcome Trust Centre for Human Genetics, University of Oxford, Oxford, United Kingdom; 2 Mammalian Genetics of Disease Unit, School of Medicine, University of Sheffield, Sheffield, United Kingdom; 3 Leeds Institute of Molecular Medicine, Wellcome Trust Brenner Building, St. James's University Hospital, Leeds, United Kingdom; 4 Mary Lyon Centre and Mammalian Genetics Unit, Medical Research Council, Harwell, United Kingdom; 5 Department of Biochemistry, University of Cambridge, Cambridge, United Kingdom; 6 Neurometabolic Unit, National Hospital, London, United Kingdom; 7 Department of Biomedical Science, University of Sheffield, Sheffield, United Kingdom; University of Washington, United States of America

## Abstract

Mutations in a number of genes have been linked to inherited dilated cardiomyopathy (DCM). However, such mutations account for only a small proportion of the clinical cases emphasising the need for alternative discovery approaches to uncovering novel pathogenic mutations in hitherto unidentified pathways. Accordingly, as part of a large-scale *N*-ethyl-*N*-nitrosourea mutagenesis screen, we identified a mouse mutant, Python, which develops DCM. We demonstrate that the Python phenotype is attributable to a dominant fully penetrant mutation in the dynamin-1-like (*Dnm1l*) gene, which has been shown to be critical for mitochondrial fission. The C452F mutation is in a highly conserved region of the M domain of Dnm1l that alters protein interactions in a yeast two-hybrid system, suggesting that the mutation might alter intramolecular interactions within the Dnm1l monomer. Heterozygous Python fibroblasts exhibit abnormal mitochondria and peroxisomes. Homozygosity for the mutation results in the death of embryos midway though gestation. Heterozygous Python hearts show reduced levels of mitochondria enzyme complexes and suffer from cardiac ATP depletion. The resulting energy deficiency may contribute to cardiomyopathy. This is the first demonstration that a defect in a gene involved in mitochondrial remodelling can result in cardiomyopathy, showing that the function of this gene is needed for the maintenance of normal cellular function in a relatively tissue-specific manner. This disease model attests to the importance of mitochondrial remodelling in the heart; similar defects might underlie human heart muscle disease.

## Author Summary

Heart disease is very common. Some cases of heart disease are strongly influenced by lifestyle and diet, whereas others have a strong genetic component. A certain form of heart failure, known as dilated cardiomyopathy (DCM) quite often runs in families suggesting that a defective gene or genes underlie this disease. We describe a new mouse mutant called “Python” which suffers from a heart disease similar to DCM. We were able to pinpoint the defective gene responsible for the disease. This gene is normally involved in the division of mitochondria, the “power plants” of the cell that generate one of the main energy supplies for the cell. This is a unique model that implicates a new gene and mechanism of disease for further investigation.

## Introduction

Idiopathic dilated cardiomyopathy (DCM) is characterised by unexplained left ventricular (LV) cavity enlargement with LV systolic impairment [1]. DCM is an important cause of congestive heart failure (CHF) with a prevalence of 36 cases per 100,000 in the United States [2]. Although the molecular pathways responsible for DCM remain largely unknown, it is estimated that between 20–50% of DCM cases are familial in nature, the large majority being inherited as an autosomal dominant trait [3]. Accordingly, the study of single gene disorders that remodel the heart to cause DCM may provide a valuable opportunity to identify critical molecules involved in disease pathways [4].

Over the past decade, DCM-causing mutations have been identified in genes encoding sarcomeric, cytoskeletal, nuclear envelope, intermediary filament, sarcoplasmic reticulum and desmosomal proteins. These findings have implicated pathogenic mechanisms whereby structural integrity, contractile force dynamics, and calcium regulation within the cardiomyocyte are perturbed. Yet such mutations only account for a minority of DCM cases [5] and many of the genes underlying DCM remain to be elucidated. A number of gene knockouts in the mouse produce features of DCM, but these phenotypes are usually recessive and so do not model the human disease. One approach that has been applied successfully to the characterisation of new disease alleles is the use of *N*-ethyl-*N*-nitrosourea (ENU) mutagenesis [6]. Treatment of mice with ENU results in a high frequency of predominantly single point mutations in the mouse germline that recapitulate the spectrum of mutations observed in many human genetic diseases. Screening of offspring reveals phenotypic variants, and the identification of the mutations underlying the abnormal phenotype can reveal new genetic regulators and novel pathways associated with disease pathogenesis. Such an approach is ‘hypothesis neutral’, making no prior assumptions about the nature of the genes or pathways involved. Using this approach we describe a novel mouse model of DCM in which a mutation in the Dynamin-1-like gene (*Dnm1l*) leads to autosomal dominant DCM and congestive heart failure.

## Results

### Python mice develop biventricular DCM

**Figure 1:**
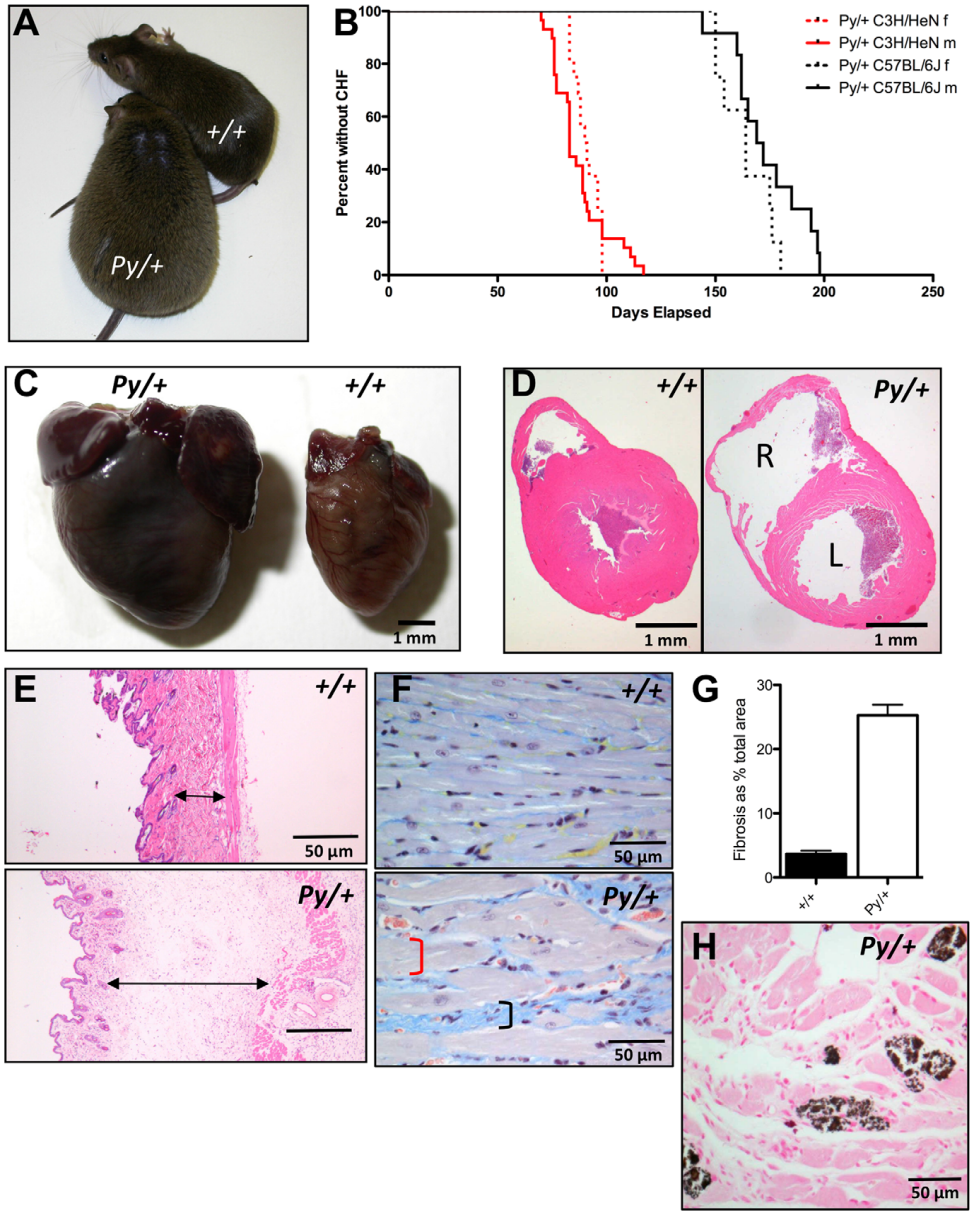
The Python mutation leads to dilated cardiomyopathy. (A) A 13-week-old Python mouse compared to a littermate control. (B) Kaplan-Meier analysis of onset of overt CHF in Python mice on the C3H/HeN and C57BL/6J genetic backgrounds compared to wild type littermate controls. (C) Photo of the excised hearts from a 13-week-old Python and wild type mouse. Note the grossly enlarged ventricles and atria. (D) H&E section through a wild type and Python heart showing showing clinical signs of CHF. Note the distended ventricles; R = right, L = left. (E) Oedematous dermal connective tissue evident in a H&E-stained section from a Python mouse showing clinical signs of CHF compared to a littermate control. (F) MSB staining of heart sections of Python mice showing clinical signs of CHF. Note enlarged cardiomyocytes (indicated by red bracket) and increased collagen deposition (indicated by black bracket). (G) Morphometric analysis of MSB-stained section demonstrating the level of increase in collagen deposition. Data shown is % of image area (mean ± SEM) that is taken up by collagen deposition from 3 *Py/+* showing overt signs of CHF and 3 *+/+* age-matched males. Five images were taken per sample. (H) Evidence of cardiac calcification in a Von Kossa-stained section of a Python heart.

The Python mouse was identified on the basis of rapid size increase, piloerection, and shallow rapid breathing in a visual screen of adult G_1_ offspring of ENU-mutagenized BALB/cAnNCrl males crossed with C3H/HeH females (Figure 1A). The phenotype was inherited in an autosomal dominant fashion with complete penetrance in both sexes. The median age of onset of overt symptoms of CHF on a C3H/HeN genetic background was 91 days for females and 83 days for males. A similar phenotype with much later onset occurred on a C57BL/6J background (median age of onset = 164 days for females, 171 days for males; Figure 1B) suggesting that strain-specific genetic modifiers influence disease onset.

The hearts of Python mice were grossly dilated by the time of overt CHF (Figure 1C). The Python hearts exhibited both biatrial and biventricular thinning and dilatation consistent with DCM (Figure 1D). The visible increase in size of Python mice was the result of substantial ascites and subcutaneous oedema (Figure 1E) accompanied on occasion by pleural effusion. In the heart, myocyte hypertrophy and interstitial fibrosis (Figure 1F) was evident. Morphometric analysis of MSB-stained sections revealed that collagenous tissue increased by almost 7-fold in hearts of terminal Python mice (Figure 1G). Prominent cardiac calcification was also evident (Figure 1H). Python mice developed CHF under specified pathogen-free (SPF) conditions, where infectious agents capable of causing myocarditis were absent. There was no microscopic evidence of myocarditis, coronary artery disease or amyloidosis, nor was there hypertrophy of pulmonary blood vessels that would indicate pulmonary hypertension. MRI analysis of embryonic hearts did not reveal any obvious anatomical abnormalities (data not shown). TUNEL staining did not reveal accelerated apoptosis in late-stage Python hearts (data not shown). We did observe hepatic congestion at the time of overt CHF, preceded by increases in the plasma levels of liver enzymes aspartate aminotransferase and alanine aminotransferase (data not shown), possibly reflecting congestive cardiac hepatopathy secondary to heart failure.

Cardiac function was analysed in male Python mice on the C3H/HeN background aged 71–78 days, i.e. approximately 2 weeks before overt clinical signs of CHF become evident. The results are summarised in Table 1. Heart rates in these conscious mice did not significantly differ between Python mice and controls, and there were no differences in any measurement of ECG interval duration (data not shown). However, LV catheterisation revealed that pressure generation was severely impaired in Python mice, with LV end-systolic pressure 22 mmHg lower than in littermate controls, and contractility 40% lower, as measured by dP/dt_max_. This reduction was not due to differences in loading conditions since dP/dt_max_ remained impaired after normalisation to instantaneous pressure. Furthermore, the Python mice had significantly elevated end-diastolic pressure commensurate with impaired contractile function. Under conditions of maximal b-adrenergic stimulation with dobutamine, Python mice had a reduced contractile reserve and a severely impaired maximum contractility. Consistent with these findings, aortic blood pressures were significantly reduced. Relaxation was also impaired in Python mice with dP/dt_min_ 46% lower than controls, and significant prolongation of the isovolumetric constant of relaxation (Tau), which is less sensitive to loading conditions. These changes occurred in the absence of LV hypertrophy or dilatation at this time point and were confirmed at post-mortem by no differences in LV or RV weights. Although at this stage lung weights were normal, indicating the absence of significant pulmonary congestion, later, at the time of appearance of overt CHF (approximately 2 weeks later) gross ascites and pulmonary congestion become rapidly manifest, consistent with the precipitous deterioration in LV function. No thrombi were observed in the atria, nor, indeed, in any heart chamber (data not shown). Finally, haematocrits were normal in all mice (data not shown), ruling out a low red blood cell count as a mechanism for LV dysfunction.

**Table 1:** Cardiovascular parameters in 11-week-old male mice.^1^

	Littermate controls (n = 6)	Python (n = 6)	*P* value
*Organ weights*			
Body weight (g)	34.0±2.8	32.5±2.1	0.31
LV weight (mg)	94±6	90±6	0.30
RV weight (mg)	28±4	27±3	0.72
Lung weight (mg)	138±5	141±16	0.65
*Echocardiography*			
End-diastolic area (cm^2^)	0.123±0.015	0.132±0.009	0.22
End-systolic area (cm^2^)	0.069±0.017	0.082±0.014	0.19
Ejection fraction (%)	44±8	38±9	0.24
Wall thickness (mm)	0.82±0.03	0.80±0.03	0.19
Heart rate (bpm)	460±52	461±59	0.98
*Haemodynamics*			
LV systolic pressure (mmHg)	97±8	75±3	0.00007
LV end-diastolic pressure (mmHg)	3.8±1.1	17.7±9.2	0.004
dP/dt_max_(mmHg/s)	8531±1084	5140±1135	0.0004
dP/dt_max_/instantaneous pressure (s^−1^)	149±8	107±23	0.002
dP/dt_min_(mmHg/s)	−8449±1381	−4564±1308	0.001
Tau (ms)	8.5±1.5	16.6±6.8	0.017
Mean arterial pressure (mmHg)	75±9	60±6	0.006
Central venous pressure (mmHg)	1.8±2.0	1.6±0.8	0.83
Dobutamine dP/dt_max_(mmHg/s)	13003±1832	6344±1424	0.00004

1 Mice were on a C3H/HeN genetic background. All data is mean ± SD with comparisons made by Student's *t*-test.

### Positional cloning of the Python mutation

**Figure 2:**
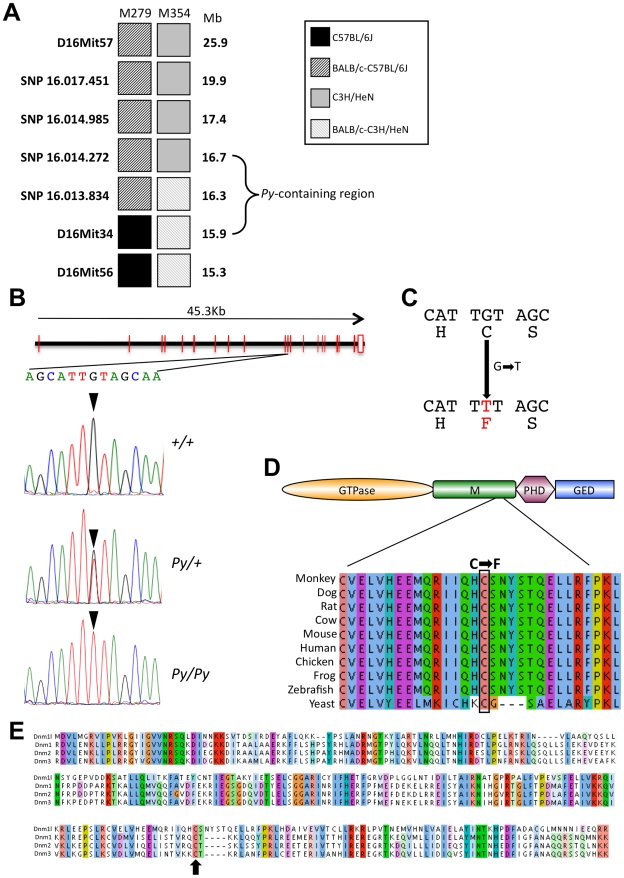
Genetic linkage analysis and positional cloning of the *Py* mutation. (A) Genotypes of two informative recombinants exhibiting the Python phenotype. The mutation was localized to a 787 Kb interval (indicated by the bracketed region). (B) Sequence of part of the 11^th^ exon of the *Dnm1l* gene reveals a G–T substitution in the Python allele. The reading frame of part of exon 11 is shown with the amino acid substitution that results. (C) ClustalW2 alignment of part of domain M of vertebrate Dnm1l orthologues and the yeast Dnm1 homologue. Note that the cysteine residue is conserved in all species examined. Amino acid colours represent similarity groupings. (D) Alignment of the entire domain M of the mouse dynamins Dnm1l, Dnm1, Dnm2 and Dnm3. Identical or functionally very similar amino acids are shown grouped together by colour on the basis of size, charge and hydropathy. The arrow indicates the position of the cysteine altered by the Python mutation.

The Python mutation was generated on a BALB/cAnN genetic background, and the original mouse exhibiting the CHF phenotype was a BALB/cAnNCrl x C3H/HeH F_1_ hybrid male. This individual was backcrossed to C3H/HeH and offspring exhibiting the CHF phenotype were examined with a panel of 53 polymorphic SNP markers spanning the entire genome at regular intervals. A strong linkage signal was observed on chromosome 16 at marker D8Mit213 (LOD score of 3.3 for a recombination fraction = 0.1). Subsequent fine mapping by backcrossing to C3H/HeH, C3H/HeN and, finally, to C57BL/6J mice narrowed the critical region containing the mutation to 787 Kb (Figure 2A). Three genes are located within this region - *Pkp2*, *Fdg4* and *Dnm1l*. Sequencing of all exons and exon-intron boundaries identified only a single mutation in Python mice; a G/C to T/A transversion in exon 11 of the *Dnm1l* gene (the official gene name as specified by the International Committee on Standardized Genetic Nomenclature for Mice but often referred to in the literature as *Drp1* or *Dlp1*) (Figure 2B). Based on the known ENU-induced mutation rate for this strain and ENU dose (1 mutation per 1.8 Mb) [7], the probability of there being an additional intronic or intergenic mutation anywhere in this region is extremely low (*P* = 0.00001) [8]. There was complete concordance between animals suffering from CHF and the Python mutation. We retrospectively regenotyped 145 DNA samples isolated from C3H and C57BL/6J Python mice that had suffered from CHF and all contained the mutation while it was absent in 189 samples from non-affected littermate control mice. None of the wild types strain examined (BALB/cAnNCrl, C3H/HeN, C3H/HeH, C57BL/6J, DBA/2J, CBA/J, 101/H, 129/S5) contained the mutation. Although *PKP2* dominant mutations have been associated with arrhythmogenic right ventricular cardiomyopathy in humans [9] an intronic or intergenic mutation is unlikely to account for the Python phenotype as all human *PKP2* mutations found to date occur in the coding region or splice sites which are not mutated in Python mice, the homozygous phenotype for the null *Pkp2* mutation is very different from the Python homozygote [10] and *Pkp2* mRNA level is not altered in Python hearts as judged by microarray analysis (data not shown) suggesting no disease-causing non-coding regulatory changes. These facts, coupled with the observation that ENU-induced mutations resulting in detectable phenotypes occur almost exclusively in the coding exons or exon-intron boundaries of genes [11], strongly suggested that this base change was the Python mutation.

The Python mutation results in the replacement of the cysteine by a phenylalanine at position 452 in the predicted Dnm1l protein (amino acid numbering according to EBI reference protein Accession No. Q8K1M6) (Figure 2C). This cysteine is located within the middle (M) domain of the protein and is fully conserved in all Dnm1l orthologues, and even the yeast dynamin homologue DNM1 (Figure 2D). The degree of evolutionary conservation of the Dnm1l protein is very high. For example, overall homology between human and mouse Dnm1l is 98%, and between zebrafish and mouse is 89%. The M domain conservation is even higher with 96% sequence conservation between mouse and zebrafish over the 291 amino acids of this domain. The cysteine residue is also conserved in the M domain of the mouse homologues of Dnm1, Dnm2 and Dnm3 (Figure 2E) despite overall homology with these domains being less than 40% (Table S1) suggesting that this cysteine plays an important role in M domain function.

### The Python mutation impairs intramolecular interaction of Dnm1l

**Figure 3:**
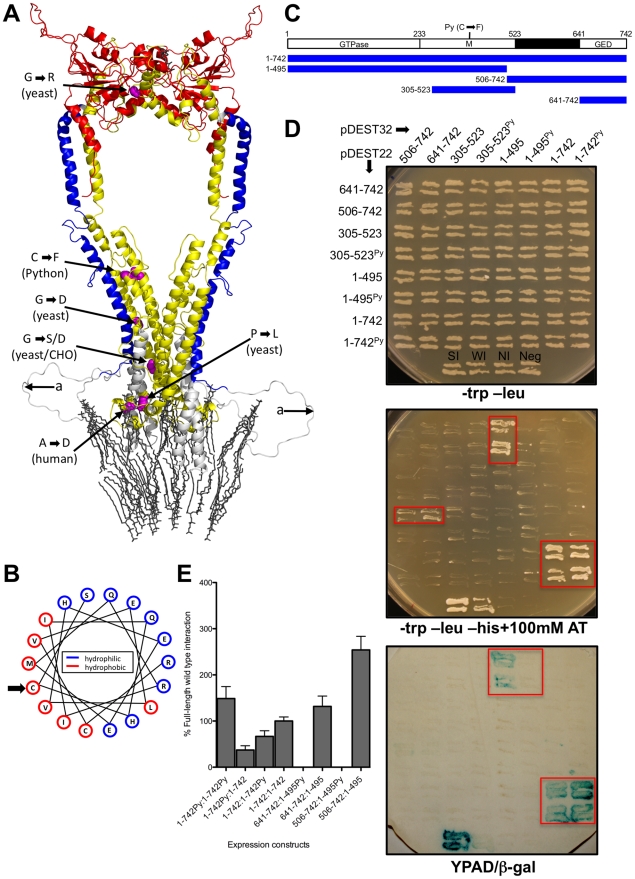
Effect of the Python mutation on the Dnm1l protein. (A) Ribbon representation of the homology model of the dimeric form of Dnm1l protein in the extended conformation and embedded in a lipid membrane. The dimeric conformation model and coordinates of the lipid membrane were generated by the superposition of the structural model of human Dnm1l onto the structure of bacterial dynamin-like protein Bdlp2 [13]. The positions of amino acid mutations reported in yeast Dnm1 and mammalian Dnm1l are shown as sphere representations in pink. A region that could not be modelled because of lack of a homologous region in BDLP is labelled with ‘a’. Colours indicate protein domains: GTPase (red), M domain (yellow) and GED (blue). (B) A helix wheel projection of the mutation-containing region of the Dnm1l protein. The hydropathy indices of the amino acids have been divided into relatively hydrophilic (blue) and relatively hydrophobic (red). An arrow indicates the cysteine that is substituted in the *Py* allele. Note how this face of the alpha-helix is relatively hydrophobic. (C) The locations within Dnm1l of the protein sequences used in the yeast two-hybrid analysis. (D) Yeast two hybrid analysis of all combinations of protein sequences as either bait (GAL4 DNA binding domain in pDEST32) and prey (GAL4 activation domain in pDEST22). Duplicate yeast colonies from each transfection grown on a –trp-leu plate (top), –trp-leu-his +100 mM AT (middle, colonies exhibiting growth are boxed in red) and a filter lift from the –trp-leu plate assayed for b-galactosidase activity (bottom). (E) Level of b-galactosidase in liquid cultures from interactors detected in (D) along with comparison to those with introduced Python mutation. Levels shown are relative to average of full-length wild type interaction. Each assay is the mean ± SEM of 6 independent measurements from 6 individual colonies for each combination.

There is no available crystal structure of any mammalian dynamin proteins but a crystal structure has been described for a bacterial dynamin-like protein. In this structure the M domain forms an elongated alpha-helical domain where the tip of the M domain helices interact with a similar region of the ‘mate’ in the dynamin homodimer [12]. Accordingly, a model of mouse Dnm1l was constructed on the basis of comparative sequence homology to the bacterial dynamin-like protein BDLP for which there is a crystal structure [12] and an electron cryomicroscopy reconstruction of BDLP assembled around a lipid tube [13]. A predicted structure could be created for most of the protein, apart from one region where there is no homology in BDLP (indicated by an ‘a’ in Figure 3A). The predicted structure of the dimeric asymmetric repeating unit in the extended confirmation (i.e. after lipid binding) is shown in Figure 3A.

There are six mutations, all dominant or semi-dominant, that have been reported in the M of domain of DNM1L or its yeast homologue DNM1–three in yeast [14], and one each in a human patient [15], a CHO cell line [16], and Python (Figure S1). These were mapped on to the predicted structure (Figure 3A). The Python mutation is located in an alpha-helix that is not predicted to affect interaction between Dnm1l monomers. However, it is located close to several other helical regions of the domain M. Furthermore, a helix-wheel projection of the region around the Python mutation-containing region predicts that one face of the predicted helix contains principally hydrophobic residues (Figure 3B). Taken together, these findings are suggestive of this face being involved in an intramolecular interaction within the Dnm1l monomer.

To test this further, we used the yeast two-hybrid assay based on GAL4 DNA binding and activation domain interactions to examine whether interactions between regions of Dnm1l could be altered by the Python mutation. We used regions of the protein that have been used by others in similar assays [17]–[22] and examined all possible reciprocal interactions of bait (in pDEST32) and prey (in pDEST22) proteins for regions of Dnm1l: full-length, N-terminal region, C-terminal region, M domain and GED (GTPase Effector Domain) (Figure 3C). On the basis of ability to grow on medium lacking histidine and b-galactosidase activity, the only strong interactions we identified were interactions between the full-length proteins, the N terminal and C-terminal regions of the proteins as reported by Zhu *et al*. [20], and the N-terminal region and the GED (Figure 3D). We found that while the Python mutation had negligible influence on the ability of the full-length proteins to interact (Figure 3D and 3E), it abrogated the ability of the N-terminal region to interact with both the C-terminal region and the GED alone (Figure 3D). Quantitative assays for b-galactosidase activity confirmed the substantial effect the Python mutation had on these interactions (Figure 3E).

### The *Dnm1l^Py^* mutation results in the impairment of mitochondrial and peroxisomal dynamics

**Figure 4:**
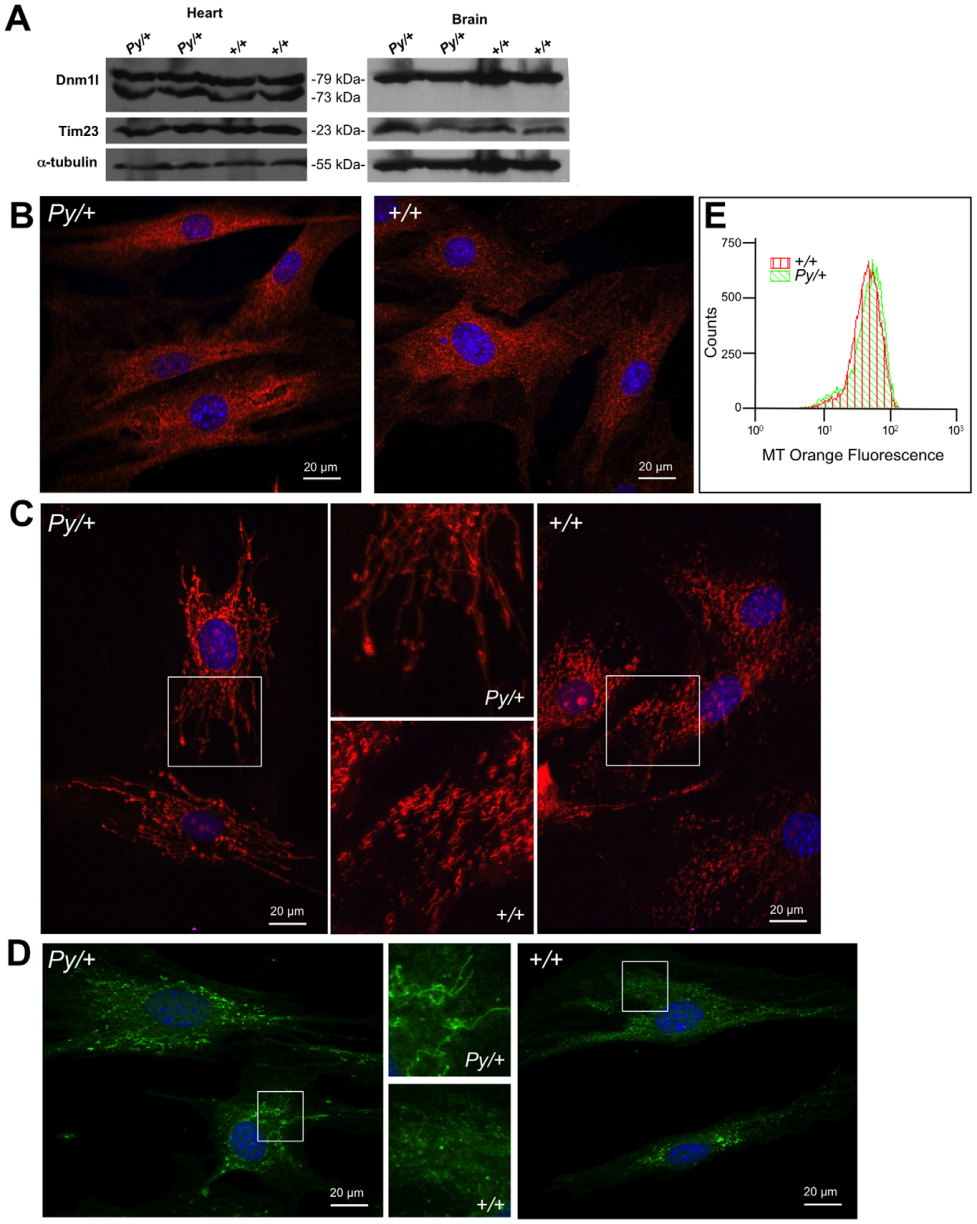
Mitochondrial and peroxisomal morphology is altered by the Python mutation. (A) Western blot analysis of total heart and liver protein extracts from 5-week-old male mice of the indicated genotypes demonstrating that Dnm1l protein levels are not altered. Western blots were re-examined using an anti-Tim23 antibody, an inner mitochondrial membrane protein, which demonstrate that the level of this mitochondrial protein is also not altered in Python mutants. After stripping, a-tubulin was detected to demonstrate loading levels of protein extracts. (B) Typical example of immunocytochemistry in neonatal mouse skin fibroblasts using an anti-DRP1 antibody. There was no appreciable difference between Python fibroblasts and wild type littermate controls. (C) Typical example of mitochondria (shown in red after staining with Mitotracker Orange) from cultured Python neonatal skin fibroblasts compared to littermate controls. (D) Typical example of peroxisomes (shown in green after incubation with an anti-catalase antibody and FITC-labelled secondary antibody) from cultured Python neonatal skin fibroblasts compared to littermate controls. The areas in C and D bounded by white squares are magnified and shown in the middle frames. Nuclei are stained with Hoechst 33258. (E) Typical example of FACS analysis of early passage skin fibroblasts from a Python neonate and littermate control after labelling with Mitotracker Orange. The histogram of fluorescence for both cells types is similar.

Given that the Python mutation occurs in a highly conserved domain of the Dnm1l protein and alters protein interaction *in vitro*, its effect on *in vivo* functions associated with Dnm1l were examined. Protein levels of Dnm1l were not altered in either heart or brain (Figure 4A) suggesting that there is no haploinsufficiency (i.e. the Python protein is assumed to be present). Dnm1l was distributed diffusely within the cell in both Python and wild type cultured skin fibroblasts (Figure 4B) suggesting that introduction of the Python protein did not drastically alter trafficking of Dnm1l. As Dnm1l function is involved in mitochondrial and peroxisomal dynamics, cultured neonatal skin fibroblasts were examined for morphology of both these organelles. Mitochondrial morphology was altered. Python mitochondria were highly elongated compared to wild type controls (Figure 4C), as were peroxisomes (Figure 4D). To determine if mitochondrial volume was altered, we utilized a novel assay where cells were loaded with a fluorescent mitochondrial marker and analyzed by flow cytometry. Intensity of fluorescence should reflect mitochondrial volume in the cell. As shown in Figure 4E, there was no difference in overall mitochondrial volume between Python and wild type fibroblasts, despite the significant changes in mitochondrial shape. This indicates that the Python mutation affects the *in vivo* functional activity of Dnm1l, thereby impairing mitochondrial fission.

### Homozygosity for the Python mutation is embryonic lethal; heterozygosity for Python modifies mitochondrial structure

**Figure 5:**
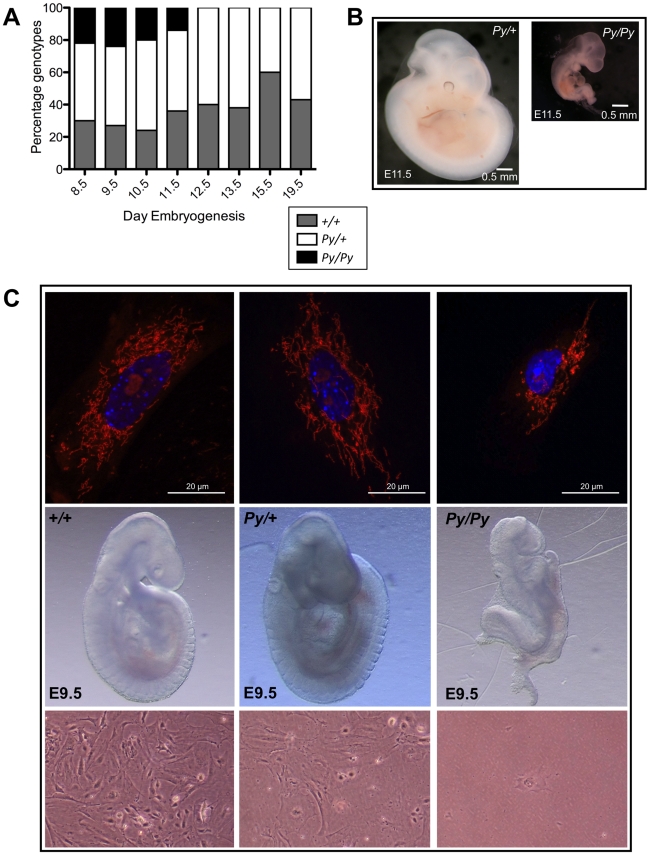
Homozygosity for the *Py* allele results in embryonic lethality. (A) Distribution of genotypes of embryos recovered from *Py/+* x *Py/+* intercrosses. Embryo numbers genotyped at each time point were: E8.5–26, E9.5–48, E10.5–20, E11.5–19, E12.5 20, E13.5–21, E15.5–10 and E19.5–14. (B) Comparison of day 11.5 embryos heterozygous and homozygous for the *Py* allele. Homozygous embryos are growth retarded and have a severe posterior truncation. (C) Comparison of day 9.5 *+/+*, *Py/+* and *Py/Py* embryos. Typical examples of individual fibroblasts obtained from culturing these embryos and stained with MitoTracker Orange are shown above. Below, typical examples of E9.5 embryonic fibroblasts in culture three days after embryo harvest. Note that the *Py/Py* cells have failed to proliferate and only a single cell is visible in this field.

To discern the functional effects of the Python mutation, we intercrossed heterozygotes to obtain homozygous animals. Genotyping of embryos demonstrated that no homozygous Python embryos could be recovered from E12.5 onwards (Figure 5A). Homozygous embryos appear to be normal up to approximately E9.5. At E11.5, *Py/Py* embryos were severely retarded in growth and exhibited a posterior truncation (Figure 5B). The homozygous embryonic phenotype is very similar to that recently reported for the *Dnm1l*-null mutation [23]–[24]. Embryos die at a similar stage and their morphology is similar. Mouse Embryonic Fibroblasts (MEFs) cultured from homozygous E9.5 embryos survived poorly in culture. Few cells attached and there was no proliferation (Figure 5C). Mitochondria of *Py/+* MEFs were abnormal with numerous long tubular mitochondria (Figure 5C), similar to *Py/+* skin fibroblasts. In contrast, homozygous Python MEFs had grossly abnormal mitochondria. Whereas a tubular mitochondrial network evenly distributed throughout the cytoplasm characterized mitochondria in *+/+* and *Py/+* MEFs, some mitochondria of *Py/Py* MEFs appeared to be spherical and aggregated (Figure 5C). This could reflect Dnm1l dysfunction alone though it may reflect a general dysfunction of these cells. As mentioned earlier, these cells fail to proliferate. Over several weeks in culture they slowly die. The nuclei staining with Hoechst 33342 demonstrated evidence of chromatin condensation in homozygous Python cells (Figure 5C), suggesting the cells could be dying by necrosis.

### The *Dnm1l^Py^* mutation results in the impairment of energy metabolism in the heart

**Figure 6:**
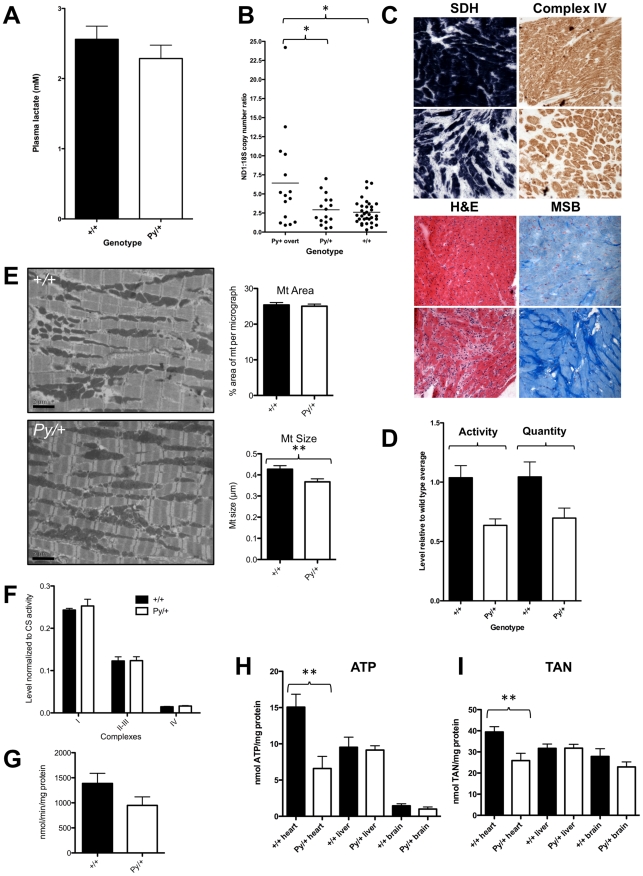
Cardiomyocyte energy metabolism is altered in Python mice. (A) Plasma lactate levels (mean ± SEM) in adult Python (n = 29) and wild type (n = 33) mice aged between 5 and 9 weeks and in mice. Sexes have been pooled. (B) The relative mitochondrial:nuclear DNA levels in heart samples from Python and control mice as assessed using Q-PCR. ‘*Py/+* overt’ refers to heart samples taken from Python mice at the time of overt symptoms of heart failure. All other mice were aged between 5 and 7 weeks at time of sampling. *P<0.05, 1-way Anova with Bonferroni's Multiple Comparison Post-Test. (C) Typical example of enzyme histochemical staining for succinate dehydrogenase (SDH) and Complex IV from hearts of Python males suffering from overt CHF (aged 93 days) and an age-matched littermate control. Typical sections stained with Haematoxylin and Eosin (H&E) and Martius/Scarlet Blue (MSB) to stain connective tissue are shown for comparison. Note the scarring and loss of myocytes in Python hearts. (D) Comparison of Complex IV activity and quantity (mean ± SEM) measured in extracts from hearts of Python mice showing overt signs of CHF and wild type controls aged 91–103 days (n = 7 Python, n = 7 controls). (E) Representative transmission electron micrographs of Python and control hearts from mice aged 9–10 weeks. Scale bar = 2 µm. The graphs on the right summarizes the morphometric measurements (mean ± SEM) of mitochondrial area (reflecting volume per cell) and mitochondrial size in Python (4 mice; n = 75 micrographs) and wild type (4 mice; n = 55 micrographs) heart EM images. **P<0.01. (F) Mitochondrial respiratory complex enzyme activities (mean ± SEM measured in extracts of heart tissue from Python (n = 8) and control mice (n = 8) aged 10 weeks and normalized to level of citrate synthase activity. All activities expressed as nmol/min/mg total protein except complex IV which is expressed as k/min/mg. Complex I: NADH: Ubiquinone reductase, Complex II–III: Succinate: Cytochrome c reductase, Complex IV: Cytochrome c oxidase. (G) Overall citrate synthase activity (mean ± SEM nmol/min/mg total protein) in heart extracts from H (n = 8 Python and n = 8 wild type, Student's t test *P* = 0.12). (H) ATP level (mean ± SEM) after normalization to total protein level in extracts of tissue from Python and control mice at age 10 weeks. Student's t test *P* values are: heart *+/+* (n = 7) vs. *Py/+* (n = 6): *P* = 0.006; liver *+/+* (n = 6) vs. *Py/+* (n = 6): *P* = 0.80; brain *+/+* (n = 6) vs. *Py/+* (n = 5): *P* = 0.29. (I) Total adenine nucleotide pool (TAN) in extracts of tissue from Python and control mice at age 10 weeks. Values shown are mean ± SEM nmol ATP + ADP + AMP normalized to mg protein. Student's t test *P* values are: heart *+/+* (n = 7) vs. *Py/+* (n = 6): *P* = 0.008; liver *+/+* (n = 6) vs. *Py/+* (n = 6): *P* = 0.97; brain *+/+* (n = 6) vs. *Py/+* (n = 5): *P* = 0.31.

The observation that Python fibroblasts exhibit abnormal mitochondria coupled with the well-recognized role of *DNM1L* in mitochondrial fission [25] and the critical role of mitochondria in both the normal function and the death of cardiomyocytes [26], led us to examine the mitochondrial and energetic phenotype of Python heterozygous mice in greater detail. Aside from the development of CHF, the Python mice did not exhibit any features prominently recognized in mitochondrial cytopathies, such as metabolic, neurological and skeletal muscle defects. They exhibited a normal general behavioral and functional profile as defined by the SHIRPA series of tests (Table S2) [27], which would reveal any major neurological abnormalities. Grip strength (a reflection of muscle strength) was normal, as was muscle histology as assessed from H&E-stained sections (data not shown). Plasma lactate levels, an indicator of general metabolic dysfunction, were not elevated in Python mice aged 5–9 weeks compared to wild type littermate controls (Figure 6A).

Given that the Python phenotype at a gross pathological level appeared to be restricted to the heart, we examined the mitochondrial phenotype of Python cardiomyocytes. There was little evidence of morphological change in Python cardiomyocyte mitochondria. For example, we examined the nuclear:mitochondrial DNA ratio in Python hearts as mtDNA is lost when *DNM1L* is down-regulated and mitochondrial fission impaired [28], However, there was no alteration in the nuclear:mitochondrial DNA ratio in Python hearts at any stage before the development of overt CHF and even at this stage only some Python hearts showed an increased ratio (Figure 6B). This suggests that a major derangement of nuclear:mitochondrial DNA ratios was not a generalized effect of the Python mutation.

There was evidence of mitochondrial function changes in hearts of Python mice suffering from overt CHF. Examination by enzyme immunohistochemistry for succinate dehydrogenase (SDH) and cytochrome c oxidase (Complex IV) activities revealed a reduction for both enzymes in Python hearts (Figure 6C). In the case of SDH there is evidence of diminished myocellular enzyme activity as indicated by less-intense staining. However for Complex IV, the enzyme immunohistochemistry suggests that in the failing heart, the change in Complex IV enzyme levels may be due to the substantial fibrosis that occurs late in the disease process rather than a change in enzyme activity. *In vitro* measurement of Complex IV activity and quantity showed that both were proportionately reduced by similar amounts in ailing Python hearts (Figure 6D), indicating that the enzyme remains fully active but is less abundant at 12 weeks of age. This reduction in overall levels at this age most likely reflects the considerable fibrosis and loss of cells that occurs in late-stage Python hearts.

Defects in mitochondrial enzyme activity are recognized as a general phenomenon in CHF [29] and, therefore, the differences observed in failing Python hearts might be secondary to the primary cause of heart failure. To determine if the Python mutation was affecting mitochondrial function prior to major changes in heart structure, we examined heart samples from Python and wild type littermates at 10 weeks of age. This is before there are any overt signs of CHF though the cardiovascular data above (Table 1) indicates that heart function is abnormal at this stage. Electron micrographs of heart samples were examined. Morphometric analysis of the proportional area occupied by mitochondria (a reflection of overall volume per cell) revealed no difference between Python hearts and controls (Figure 6E). This in agreement with the flow cytometry findings in Python skin fibroblasts (Figure 4E). Nor were myofibre widths significantly different between Python hearts and controls (data not shown). There was no evidence of the membrane pinching reported in the *Dnm1l*-null mouse fibroblasts [Ishihara2009], nor was there evidence of large aggregates as has been reported for *in vitro* cultured *DNM1L* mutants [30]. However, the average size of a mitochondrion was slightly smaller in Python hearts than wild type hearts (Figure 6E). Smaller cardiomyocyte mitochondria have been previously reported in some cases of heart failure [31]–[33]. In the case of Python, if mitochondrial volume is not altered but mitochondrial tubules are extended in length, then a smaller cross-sectional transverse area of mitochondria would be consistent with this.

If Dnm1l affects mitochondrial dynamics, we predicted that the end point of this would be impairment of respiratory chain function. Examination of mitochondrial enzyme complex activities normalized to citrate synthase activity in heart samples from 10-week-old mice revealed no differences (Figure 6F and 6G). There was a slight reduction in the overall level of mitochondrial citrate synthase activity in Python hearts at this age but it was not statistically significant (Student's t test, *P* = 0.11) (Figure 6G) also suggesting that the total mitochondrial volume in Python hearts was not reduced. As the end-point of respiratory chain function is ATP synthesis, and given that down-regulation of *DNM1L* results in a reduced rate of ATP synthesis [28], [34], myocardial ATP and total adenine nucleotide (TAN) levels were measured using HPLC. Python hearts exhibited a dramatic, approximately 50%, reduction in ATP and TAN levels (Figure 6H and 6I) compared to hearts from littermate controls. In liver and brain at the same age, ATP and TAN levels were similar in Python mice and controls (Figure 6H and 6I), indicating that the defect in ATP generation was not a general one. These results were confirmed by using complementary quantitative bioluminescence assays (Figure S2).

A range of metabolites in the heart was examined using high-resolution ^1^H NMR spectroscopy and Gas Chromatography Mass Spectrometry (GC-MS)-based metabolomic approaches [35] on Python and control mice. The metabolite changes identified are summarized in Table 2. Significant reductions in Python hearts were noted in mitochondrial metabolic intermediates or accessory molecules e.g. succinate, malate, fumarate, pyruvate (all associated with the citric acid cycle), creatine, glucose, AMP and adenosine. Two notable increases were in the amino acids glycine and proline. These account for approximately 50% of the amino acids in collagen, possibly reflecting the fibrosis in Python hearts.

**Table 2:** Changes in metabolite levels measured in hearts from Python mice and littermate controls.^1^

^1^H NMR spectra	Metabolite	Python∶wt ratio	*P* value (t test)
Increased in Python	Aspartate	2.11	4.2×10^−9^
	Glutamate	1.15	5.1×10^−4^
	Glycine	1.67	4.8×10^−15^
	Proline	1.24	1.4×10^−8^
	Valine/leucine/isoleucine	1.69	6.3×10^−10^
Decreased in Python	Choline	0.80	1.6×10^−4^
	Creatine	0.69	2.3×10^−11^
	Lactate	0.80	2.6×10^−5^
	Succinate	0.58	3.2×10^−8^
	Taurine	0.92	1.1×10^−3^

Gas chromatography-mass spectrometry			
Increased in Python	Asparagine	1.62	2.3×10^−4^
	Glycine	1.57	1.3×10^−8^
	Proline	2.35	3.1×10^−5^
	Threonine	1.82	3.3×10^−6^
	Uric acid	1.65	4.4×10^−4^
Decreased in Python	α-glycerophosphoric acid	0.69	3.5×10^−7^
	Adenosine	0.37	7.0×10^−7^
	AMP	0.27	5.7×10^−7^
	Creatine	0.44	1.5×10^−5^
	Fumarate	0.55	5.9×10^−5^
	Glucose	0.42	1.1.×10^−4^
	^1^H-Indole-2,3-dione	0.37	2.1×10^−6^
	^9^H-Purine-6-amine	0.66	5.0×10^−4^
	Malate	0.59	2.5×10^−6^
	Myo-inositol	0.66	1.1×10^−5^
	Pyruvate	0.47	3.8×10^−7^
	Ribose	0.40	1.4×10^−3^
	Succinate	0.54	5.9×10^−5^

1 Mice were on a C3H/HeN genetic background. Average of individual measurements from each heart sample of mice aged 10–11 weeks (n = 10 *Py/+*; n = 10 *+/+*).

## Discussion

We report the identification, through ENU mutagenesis, of a novel genetic cause of cardiomyopathy. Our principal finding is that a missense mutation in the middle domain of *Dnm1l*, whose product is critically involved in mitochondrial fission, results in DCM. The resulting defect in mitochondrial remodelling renders the Python hearts progressively energy deficient potentially contributing to the phenotype [36].

The fundamental importance of mitochondrial remodelling in mammalian pathophysiology has been underlined by *in utero* lethality and cerebellar degeneration in mice with homozygous mutations in *Dnm1l* itself, as well as *Mfn1*, *Mfn2*, or *Opa1* [23], [37], [38]. Given the heart's manifest dependency on mitochondria [39] as evidenced by its frequent involvement in mitochondrial disorders [40], mitochondrial remodelling defects might be expected to occur in some forms of myocardial disease. Recently it has been reported that mitochondria are smaller in failing hearts and DNM1L protein levels were increased in DCM heart samples [33] but until now, there has been no direct evidence that genes involved in regulating mitochondrial dynamics might be involved in heart failure. Python is the first such example.

DNM1L is a member of the dynamin superfamily. In the higher order spirals formed by dynamin, the basic repeating unit appears to be a dimer [41]. The association between the GED and M domains forms a ‘stalk’ conformation through which strong inter-molecular interactions of the dimers occur [41]. Mutations in the M domain have accordingly been shown to adversely affect self-assembly into a higher order oligomeric structures that are critical to Dnm1l function [22] and conformational changes within this region are associated with the constriction of dynamin tubes that facilitates fission [42]. It is not difficult to envisage how the substitution of a bulky hydrophobic phenylalanine residue into a helix-rich region of the M domain (Figure 3A) has the potential to modify interactions necessary for Dnm1l's effective function. As a corollary, our yeast two hybrid analysis showed Python's capacity to abrogate Dnm1l's N-terminal region interacting with the C-terminal region (and GED), while leaving the interaction between Dnm1l monomers unaffected. A similar effect was reported when the S637D mutation was introduced into Dnm1l [43]. This effect of the mutation on Dnm1l higher order structure is consistent with a dominant-negative mode of action. Since there appears to be no change in overall Dnm1l protein levels, we propose that the Python monomer is readily incorporated into dimers with the wild type protein but fails to function effectively within that dimer due to defective intramolecular interactions. If this model is accurate, only 1 in 4 Dnm1l dimers might be expected to be fully functional.

This study cannot exclude a systemic (i.e. non-cardiac) impact of the C452F mutation, though from a clinical perspective, most tissues and organ functions appeared to be grossly spared. The liver was the one organ that did show evidence of extra-cardiac involvement but this might reflect congestive cardiac hepatopathy caused by heart disease. The predominant cardiac phenotype in the Python heterozygotes contrasts with the marked skeletal muscle and metabolic abnormalities in the infant with a dominant mutation in *DNM1L* [15]. It is unclear why the Python mutation manifests an overt phenotype only in heart. The reported human mutation in domain M, a dominantly acting alanine to aspartic acid mutation, appears to be much more severe than the Python mutation appeared to have a widespread metabolic defect. This may be related to the location of the amino acid within the structure and/or the chemical nature of the amino acid substitutions involved. An inter-species difference in DNM1l functionality is possible but unlikely given the extremely high degree of inter-species conservation. All 7 mutations identified in the M domain that affect mitochondrial dynamics are conserved not only in the M domain of DNM1L orthologues across multiple species (Figure S1), but are also conserved across the M domain of DNM1, DNM2, DNM3 and DNM1L (Figure 2E). The human alanine to aspartate mutation occurs in a region of the protein that might interact with the membrane (Figure 3A). When overall compatibility of the amino acid replacements are compared the Python amino acid replacement scores significantly higher in compatibility with its original amino acid in terms of hydrophobicity, size and charge compatibility (15.8, 9.4, 18.17 for the Python mutation compared to 12.8, 8.6 and 12.82 for the A400D human DNM1L mutation) [44]. Given that there is no evidence that this cysteine is involved in disulphide bonding in DNM1L, and its effect in the yeast two-hybrid system on intramolecular but not intermolecular Dnm1l interactions, its effect on Dnm1l function may be relative mild, still enabling the formation of dimers but with reduced functionality.

Although the gross impact of the heterozygous C452F Dnm1l mutation on cardiac morphology was relatively subtle at ∼70 days, the corresponding functional and, even more so, the biochemical phenotypes were apparent. This was reflected in uniform lethality shortly thereafter. The degree of ATP deficiency in the Python hearts at this age is, to the best of our knowledge, unprecedented. The apparent fall of ATP levels to 44% of wild type levels in hearts from 10-week-old mice (15.1 vs. 6.6 nmol ATP/mg/protein in *+/+* and *Py/+* hearts at 10 weeks of age confirmed by two independent means) may have been exaggerated by increased fibrosis, yet still seems beyond the range by which it is thought that cardiac [ATP] is allowed to diminish (∼25% to 30%) even in CHF [39]. We used an exacting method involving HPLC to measure these levels to ensure they were as accurate as possible. However a major caveat to these observations, is that the degree of difference in ATP levels observed herein will inevitably be exaggerated by the technical limitations associated with measuring ATP levels in tissue samples after excision from the body. The resulting rapid depletion of ATP pools may have precluded accurate measurements. However, a difference in ATP levels between Python and control hearts (that is not found in liver and brains) may indicate that the Python mutation does espouse an aberrant and progressive impact on myocardial energetics.

One possible explanation for the Dnm1l-mediated cardiomyopathy is cardiac energy deficiency. It is recognized that CHF is associated with, and in many cases exacerbated by, cardiac energy deficiency [36], [39], [45], [46]. Indeed, the commonality of CHF to numerous primary mitochondrial diseases represents an excellent source of evidence that primary energy deficiency can and does cause CHF [36], [47]. As reduction of Dnm1l function is known to impair cellular energetics [28], [34] and as this pattern of cellular energetic impairment is profoundly and progressively manifested in Python hearts even in advance of gross cardiac dysfunction, cardiac energy deficiency may contribute to the phenotype of Python hearts and may be the proximate cause.

Detailed questions remain regarding the impact of the C452F substitution. Further biochemical and cellular studies are needed to investigate the hypothesis that such M domain mutations alter the intra/inter-molecular interactions and alter the homo-oligomerisation properties of Dnm1l [48]. Such studies will have to explain why in the context of the subtle reticular mitochondrial changes in Python, there is a discrepancy between the lack of an overall decrease in mitochondrial volume or function and the degree of ATP depletion. There are a number of other metabolic and cellular processes that could be altered by the Python mutation. Calcium cycling, for example, is altered in cells lacking the mitochondrial fission protein Mitofusin 2, reflecting its role in endoplasmic reticulum (ER)-mitochondria tethering [49]. Inhibition of *Dnm1l* alters ER structure [50], though we could find no alteration in the morphological appearance of the ER in Python fibroblasts (data not shown). Nevertheless, further investigation of Ca^2+^ uptake into mitochondria and release from ER is warranted. An energy defect not reflected in altered respiratory complex enzyme activity might also result from other disturbances such as uncoupling of electron transport and ATP production, perturbation of supercomplexes [51], cell cycling [52] organelle quality control through autophagy, or generation of reactive oxygen species [28]. It is possible that Python has effects on Dnm1l function, unrelated to mitochondrial dynamics.

The tissue specificity of the Python defect warrants further investigation. This may reflect unique properties of cardiomyocytes, their mitochondria, or a unique role for *Dnm1l* in cardiomyocytes. For example, a role for DNM1L in the heart has been inferred by Ong *et al.* who observed that mitochondrial fission protects the heart against ischemia/reperfusion [53]. One strategy to address this question would be to effect conditional inactivation of *Dnm1l* in cardiomyocytes, in a similar manner to mitochondrial fusion factor elimination in skeletal muscle [54]. In conclusion, we report the first model of mitochondrial remodeling to be associated with cardiomyopathy. It is likely that the C452F substitution in the M domain of Dnm1l alters the balance of mitochondrial fission and fusion. The impairments in mitochondrial remodeling and function result in a relatively tissue specific disease as manifested by rapidly progressive cardiomyopathy. It is plausible that mutations in *Dnm1l* that are similarly subtle and hence do not represent a barrier to viability, will be identified and prove to be of importance in human disease.

## Materials And Methods

### Mice

Mice were maintained in high health status facilities with access to food and water *ad libidum*. All work was approved by the Animal Ethical Review Committees of MRC Harwell, University of Sheffield and University of Leeds, and the UK Home Office, and conducted with the highest quality of animal care and in accordance with the 3Rs.

### ENU mutagenesis

Adult BALB/cAnNCrl mice aged 10 weeks were mutagenized by intraperitoneal injection of two weekly doses of 100 mg/kg ENU, mated with C3H/HeH and F_1_ offspring screened for abnormalities. Python mice were further backcrossed to C3H/HeN and C57BL/6J. All tissue samples used for metabolic and cellular analysis were derived from a line generated by inbreeding of an N_1l_ backcross to C3H/HeN.

### Genetic mapping

Genome-wide low-resolution mapping was performed using DNA samples from 15 N_2_ C3H/HeH backcross animals that were identified as carriers based on the development of congestive heart failure. Genomic DNA samples isolated from tail biopsies of these animals were screened by PCR amplification and gel electrophoresis with 53 microsatellite markers spaced at regular intervals across the genome. Samples were genotyped as either homozygous C3H or heterozygous BALB/c-C3H for each marker. For finer mapping, crosses of Python mice with both C3H and C57BL/6J were used, and further microsatellite markers polymorphic between either BALB/c and C3H or BALB/c and C57BL/6J were used to identify Python mice that were recombinant in the critical region. Single nucleotide polymorphisms (SNPs) were genotyped by sequencing of PCR products amplified with primers flanking the SNP site. Sequencing of candidate genes involved designing primer pairs to amplify individual exons as well as flanking splice donor/acceptor sequences. All exons and splice sites in the critical region were sequenced using homozygous as well as heterozygous Python DNA, ensuring that no base changes were missed.

### Measurement of blood lactate

A small drop of blood was obtained from the lateral tail vein and processed using a Lactate Pro analyzer (HabDirect, Southam, UK).

### Histology and TEM

Tissues for light microscopy were emersion fixed in 10% neutral buffered formalin and wax-embedded 3 µm sections were stained with haematoxylin and eosin or Mauritius Scarlet Blue (MSB). Mice were perfusion fixed for TEM. *In situ* staining for succinate dehydrogenase and Complex IV activities were a previously described [47], [55], [56].

### *In vivo* cardiac phenotyping

Conscious ECG measurements were obtained in unrestrained male Python mice and littermate controls (n = 6 of each) at 10 weeks of age using the non-invasive AnonyMOUSE ECG screening tool (Mouse Specifics Inc). One week after, at 75±2 days of age, the same mice were anaesthetised with isoflurane and placed on a homeothermic blanket. Parasternal short- and long- axis views were obtained under 1.25% isoflurane anaesthesia using an Agilent Sonos 5500 with 15 MHz transducer. The LV was cannulated via the right carotid artery with a 1.4F Mikro-tip conductance cannula (SPR-839, Millar Instruments). The superior vena cava was cannulated with a second 1.4F Millar cannula (SPR-671) to measure central venous pressure. Mice were allowed at least 15 minutes equilibration before baseline aortic and ventricular pressure measurements were obtained. Dobutamine was given by intraperitoneal injection (1.5 µg/g body weight) and pressure measurements obtained under maximal b-adrenergic stimulation. Mice were then killed by cervical dislocation and organs washed in heparinised saline, blotted and weighed. Two experiments were performed daily, alternating between genotypes for morning and afternoon experiments.

### Enzymatic assays

All activities were determined at 30°C. Prior to analysis cells were subjected to three cycles of freezing and thawing to lyse membranes. Enzyme activities were assessed were assessed using a Uvikon 940 spectrophotometer (Kontron Instruments Ltd, Watford, UK). Complex I activity was measured according to the method of Ragan e*t al*. [57]. Complex II-III activity was measured according to the method of King [58]. Complex IV activity was measured according to the method of Wharton and Tzagoloff [59]. Citrate synthase (CS; EC 1.1.1.27) activity was determined by the method of Shepherd and Garland [60]. Enzyme activities were expressed as a ratio to citrate synthase to compensate for mitochondrial enrichment in cell samples [61]. Measurement of Complex IV activity and quantity in terminal heart samples used a Complex IV Mouse Duplexing (Activity + Quantity) Microplate Assay Kit (Mitosciences, Eugene, OR) according to the manufacturer's instructions.

### NMR and mass spectroscopy

Tissues were extracted using a methanol: chloroform: water extraction procedure to separate aqueous soluble metabolites from lipids as previously described [62]. Briefly, ∼100 mg tissue were pulverised with dry ice. 600 µl methanol: chloroform (2∶1) was added and the samples were sonicated for 15 min. Water and chloroform were added (200 µl of each). The resulting aqueous and organic layers were separated from the protein pellet. The organic layer was dried overnight in a fume hood whilst the aqueous extracts were evaporated to dryness using an evacuated centrifuge (Eppendorf, Hamburg, Germany).

#### NMR spectroscopy

The aqueous extracts were rehydrated in 600 µl D_2_O, buffered in 0.24 M sodium phosphate (pH 7.0) containing 1 mM (sodium-3-(tri-methylsilyl)-2, 2, 3, 3-tetradeuteriopropionate (TSP) (Cambridge Isotope Laboratories Inc., Andover, MA, USA). The samples were analysed using an AVANCE II+ NMR spectrometer operating at 500.3 MHz for the ^1^H frequency (Bruker Gmbh, Germany) using a 5 mm ATMA TXI probe. Spectra were collected using a solvent suppression pulse sequence to saturate the residual ^1^H water proton signal (noesypr1d pulse sequence; relaxation delay = 2 s, t_1_ = 3 µs, mixing time = 150 ms, solvent presaturation applied during the relaxation time and the mixing time). 128 transients were collected into 16 K data points over a spectral width of 12 ppm at 27°C.

#### GC-MS

Aqueous samples were derivatised using the procedure reported [63]. 150 µl of the D_2_O sample used for ^1^H NMR spectroscopy was evaporated to dryness in an evacuated centrifuge and 30 µl methoxyamine hydrochloride (20 mg ml^−1^ in pyridine) was added. The samples were vortex mixed for 1 minute, and derivatised at room temperature for 17 hours. Samples were then silylated with 30 µl of *N*-methyl-*N*-trimethylsilyltrifluoroacetamide (MSTFA) for 1 hour at room temperature. The derivatised samples were diluted (1∶10) with hexane prior to GC-MS analysis. Organic phase metabolites were derivatised by acid catalyzed esterification [64]. Lipids were dissolved in 0.25 ml of chloroform/methanol (1∶1 v/v). 0.10 ml BF_3_/methanol (Sigma-Aldrich) was added and the vials were incubated at 80°C for 90 minutes. Once cool 0.3 ml H_2_O (mQ) and 0.6 ml hexane was added and each vial vortex mixed for one minute. The aqueous layer was discarded and the remaining organic layer was evaporated to dryness before reconstitution in 200 µl hexane for analysis. The derivatised aqueous samples were injected into a Thermo Electron Trace GC Ultra equipped with a 30 m×0.25 mm ID 5% phenyl polysilphenylene- siloxane column with a chemically bonded 25 µm TR-5MS stationary phase (Thermo Electron Corporation; Injector temperature = 220°C, helium carrier gas flow rate = 1.2 ml min^−1^). The initial column temperature was 70°C; this was held for 2 min then increased by 5°C min^−1^ to 310°C and was held for 20 min. The derivatised organic metabolites were injected onto a ZB-WAX column (30 m×0.25 mm ID×0.25 µm df; 100% polyethylene glycol). The initial column temperature was 60°C; this was held for 2 min then increased by 10°C min^−1^ to 150°C and then by 4°C min^−1^ up to a temperature of 230°C where it was held for 7 min. The column eluent was introduced into a DSQ quadrupole mass spectrometer (Thermo Electron Corporation) (transfer line temperature = 310°C for aqueous metabolites and 240°C for lipid metabolites, ion source temperature = 250°C, electron beam = 70 eV). The detector was turned on after a solvent delay of 240 s and data was collected in full scan mode using 3 scans s^−1^ across a mass range of 50–650 m/z.

#### Data Analysis

NMR spectra were processed using ACD SpecManager 1D NMR processor (version 8, ACD, Toronto, Canada). Spectra were Fourier transformed following multiplication by a line broadening of 1 Hz, and referenced to TSP at 0.0 ppm. Spectra were phased and baseline corrected manually. Each spectrum was integrated using 0.04 ppm. integral regions between 0.5–4.5, and 5.1–10.0 ppm. To account for any difference in concentration between samples each spectral region was normalised to a total integral value of 10000. GC-MS chromatograms were analysed using Xcalibur, (v. 2.0, Thermo Fisher Corp), integrating each peak individually. Peaks were normalised so that the total sum of peaks was set to 10000. Deconvolution of overlapping peaks was achieved by generating traces of selected ions. A 0.1 minute threshold window was used for the deviation of peaks away from the predicted retention time across the data set. Structures were assigned using both the NIST database of mass spectra and analysis of standard compounds. Datasets were imported into SIMCA-P 10.0 (Umetrics, Umeå, Sweden) for processing using PCA, PLS (Partial least squares, a regression extension of PCA used to separate out a trend from other variation in the data set) and PLS-DA (a regression extension of PCA used for classification). Identification of major metabolic perturbations within the pattern recognition models was achieved by analysis of corresponding loadings plots. Additionally, R^2^ and Q^2^ were used as measures for the robustness of a pattern recognition model. R^2^ is the fraction of variance explained by a component, and cross validation of R^2^ gives Q^2^ which reveals the fraction of the total variation predicted by a component. Both values are indicative of how good the overall model is. Typically a robust model has R^2^>0.50 and Q^2^>0.40. Coefficient scores rank the observations according to their contribution to the model. In order to confirm which metabolites contributed significantly to each model, each variable was assessed by a jack-knifing routine to assess its contribution to a given component. Only variables deemed to have a coefficient significantly different from zero were included.

### Quantification of mtDNA and 18S rRNA by Q-PCR

100,000 cells were resuspended in 100 µl extraction solution (0.2 mg/ml proteinase K, 0.2% SDS and 5 mM EDTA in PBS) and incubated at 50°C for 3 h. Total DNA was then precipitated by addition of 10 µl of 3 M sodium acetate (pH 5.2), 110 µl isopropanol and incubation for 20 minutes on ice before centrifugation at 12,000 rpm at 4°C. The DNA-pellet was washed once with cold 70% ethanol, air dried for 15 min and resuspended in 100 µl TE buffer at 4°C overnight. Realtime PCR amplification was performed on 10 ng of total DNA using a iCycler (Bio Rad) and iQ SYBR Green Supermix (BioRad) following the manufacturer's instructions. A 211 bp fragment of the mtDNA 12S RNA gene was amplified between nucleotide 1095 and nucleotide 1305 (Forward primer: 5′ GCTCGCCAGAACACTACGAG 3′, reverse primer: 5′ CAGGGTTTGCTGAAGATGGCG 3′). Elongation translation factor 1 gene (EEF1A1) was used as an endogenous reference across all experimental conditions (Forward primer: 5′ GGATTGCCACACGGCTCACATT 3′, reverse primer: 5′ GGTGGATAGTCTGAGAAGCTCTC 3′).

### Yeast two-hybrid analysis

Regions of *Dnm1l* were amplified from a mouse IMAGE cDNA clone plasmid and cloned into the vectors pDEST22 (prey) and pDEST32 (bait) (Invitrogen Ltd., Paisley, UK) according to the manufacturer's instructions. The two hybrid tests were performed in the yeast strain MaV203, which contains a HIS3 promoter driving expression of *HIS3* and a *GAL1* promoter to drive expression of *LACZ* as chromosomally integrated reporter genes. Interactions were initially tested by plating on yeast dropout medium agar without leucine, tryptophan and histidine and with increasing amounts of 3-amino-1,2,4-triazole (10, 25, 50 and 100 mM). Interactions were then further tested using a semi-quantitative b-glactosidase filter lift assay followed by a quantitative b-galactosidase liquid culture assay according to the Pro-Quest instruction manual (Invitrogen Ltd., Paisley, UK).

### Isolation of embryonic fibroblasts

For skin fibroblasts, 2–4-day-old pups were humanely culled, and a portion of the skin removed, washed in PBS and finely minced using a razor blade. A small piece of tissue was retained for genotyping purposes. Numerous small segments of tissue approximately 1 mm^3^ were placed well spaced on 10 cm Petri dishes and allowed to air dry for 10 minutes, DMEM containing Glutamax and 10% FCS (Invitrogen Ltd., Paisley, UK) was then carefully added to the dish so as to avoid dislodging the tissue pieces. Plates were cultured for 7 days at 37°C, 5% CO_2_, then outgrowing cells were harvested with trypsin and passaged in the same medium. For embryonic fibroblasts, embryos were dissected from the uterus and the yolk sacs were removed for use in genotyping the embryos. The embryos were placed in 50 µl 0.05% trypsin and macerated using a 20 µl disposable pipette tip. After incubation for 15 minutes at 37°C, the cells were counted and plated in one well of a 6-well plate in DMEM containing Glutamax and 10% FCS and cultured at 37°C, 5% CO_2_.

### Labeling of mitochondria in fibroblasts

For microscopy, fibroblasts were cultured on glass coverslips in 50–200 nM Mitotracker Orange CM-H_2_TMRos in DMEM containing Glutamax and 10% FCS for 45 minutes at 37°C. Cells were washed in PBS, then fixed in 4% paraformaldehyde for 20 minutes at room temperature. Cover slips were mounted on glass slides in a drop of ProLong Gold mounting medium (Invitrogen Ltd., Paisley, UK) containing 10 µg/mL Hoechst 33258. For FACS analysis, cells were harvested by trypsinization, washed with PBS, then fixed for 20 minutes at 4°C in 4% paraformaldehyde. Cells were subsequently centrifuged and resuspended in PBS.

### Western blot analysis

Brain and heart samples from 5-week-old mice were snap frozen in liquid nitrogen then homogenized in RIPA (50 mM Tris-HCl pH 8, 150 mM NaCl, 1% NP-40, 0.5% sodium deoxycholate, 0.1% SDS) containing 1× protease inhibitor cocktail (P8340; Sigma-Aldrich, Dorset, UK). Equal amounts of protein were separated by Laemmli stacking/separating SDS PAGE and blotted to Hybond-P (GE Healthcare, Little Chalfont, UK), then individual proteins were detected using antibodies to Drp1 (Clone 8; BD Biosciences, Oxford, UK), Tim23 (Clone 32; BD Biosciences, Oxford, UK), and a-tubulin (T6074; Sigma-Aldrich, Dorset, UK). An anti-mouse HRP-conjugated antibody (7076; Cell Signaling, NEB, Hitchin, UK), was used for detection in combination with ECL Western Blotting Detection Reagents (GE Healthcare, Little Chalfont, UK).

### Immunocytochemistry

For peroxisome labeling, a primary rabbit anti-catalase IgG (ab16731, Abcam) was used with a goat anti-rabbit FITC-conjugated IgG (ab6717, Abcam). For examining Dnm1l distribution in fibroblasts, an anti-Drp1 primary antibody (Clone 8; BD Biosciences, Oxford, UK) with a Daylight549-conjugated anti-mouse Ig was used.

### Fluorescent imaging

All images were taken on a Zeiss AxioCam MRc5 microscope using Axiovision Release 4.7.2 software. For imaging mitochondria with Mitotracker Orange and Dnm1l using a Daylight549-labelled secondary antibody, a filter with excitation 530–585 nm, emission 600–660 nm was used. For Imaging peroxisomes with a FITC-labeled secondary antibody, a filter with an excitation of 450–490 nm, emission 510–560 nm was used. For FACS analysis, cells were incubated in 200 nM Mitotracker Orange CM-H_2_TMRos in DMEM containing Glutamax and 10% FCS for 45 minutes at 37°C, harvested using trypsin, washed with PBS and fixed by incubation in 4% paraformaldehyde for 20 minutes at 4oC. Cells were washed in PBS and resuspended in PBS containing 1% FCS and analyzed for fluorescence on a CyAnADP O2 flow cytometer (Dako). The gate for Mitotracker Orange-positive cells was set using control cells that were not labelled.

### ATP and total adenine nucleotide pool measurements

Hearts were excised, washed in heparinised normal saline, blotted and weighed, before being snap frozen in liquid nitrogen and stored at −80°C. Total adenine nucleotide (TAN) content and myocardial adenosine triphosphate (ATP) were measured by High Performance Liquid Chromatography (HPLC) as previously described [65]. For the purpose of calculating activity per mg of protein, the Lowry method was used.

### Homology modelling

The structure of the human DNMlL protein (Uniprot [66] accession number O00429) was predicted by homology modelling as follows. The structure of the bacterial dynamin-like protein BDLP [13] (PDB [67] identification code 2w6d) was used as template. Given the low sequence identity (around 12%), the alignment between target sequence and template was performed using a profile-to-profile alignment method implemented in the FFAS03 server [68] (http://ffas.ljcrf.edu). Profile-to-profile alignments are superior in terms of sensitivity and alignment quality compared to traditional pair wise alignments (and in particular at low sequence identity). The FFAS03 score for the alignment was −33.5 implying significant similarity (FFAS03 scores below −9.5 reported less than 3% false positive under benchmarking conditions [68]). The alignment was manually inspected and the structural model was derived using MODELLER [69] as previously described [70]. Structure representations were prepared using the molecular visualization program PyMOL (http://www.pymol.org).

### Software and databases

DNA sequence alignments for detecting mutations were performed using DNASTAR (DNASTAR Inc. Madison, WI). Protein alignments were performed using CLUSTALW2 and ALIGN software available online from the European Bioinformatics Institute (www.ebi.ac.uk). Prism software (GraphPad Software, Inc., La Jolla, CA) was used throughout for statistical analysis. Morphometric analysis used ImageJ [71]. For measurement of mitochondrial area and size, contrast of micrographs was enhanced by 0.5%. After setting the scale, the threshold was altered such that only mitochondria were marked. The thresholded image was then analyzed for area, and area fraction for particles greater than 50 pixels. For measurement of area stained in MSB sections, the image type was set to RGB stack and the threshold set to detect areas positive for collagen. The thresholded image was then analyzed for area above threshold.

## Supporting Information

Figure S1 Alignment of domain M in multiple species and mutations found in Dnm1l protein homologues that have an effect on mitochondrial morphology. Identical or functionally very similar amino acids are shown grouped together by colour on the basis of size and hydrophobicity. Reported mutations that result in amino acid substitutions are 1, G269R yeast [14]; 2, G385D yeast [14]; 3, G398S yeast [14]; 4, G369D hamster CHO cell line [16]; 5, A400D human [15]; 6, P444L yeast [14]; 7, C452F mouse (Python).
(9.96 MB TIF)

Figure S2 ATP measurements (mean plus/minus SD) in heart and brain samples expressed as level relative to wild type mean, measured using a quantitative bioluminescent method. Tissue samples were minced and then digested for 45 minutes in 10 mM Tris pH 8.0 and 100 µg/ml proteinase K at 55°C. Protein concentrations were estimated using a Bio-Rad DC Protein Assay kit according to the manufacturers instructions. Samples were then normalized based on total protein. ATP was measured using an ATP Luminescence Assay Kit (Invitrogen Corp.) according to the manufacturers instructions.
(0.71 MB TIF)

Table S1 Degree of similarity of domain M of mouse dynamin proteins.
(0.03 MB DOC)

Table S2 SHIRPA Assessment of Python mice.
(0.04 MB DOC)
