# Supplementary figures and images for: A Mutation in the Mitochondrial Fission Gene Dnm1l Leads to Cardiomyopathy
Source: PLoS Genet. 2010 Jun 24;6(6):e1001000. doi: 10.1371/journal.pgen.1001000 (PMC2891719; doi:10.1371/journal.pgen.1001000)

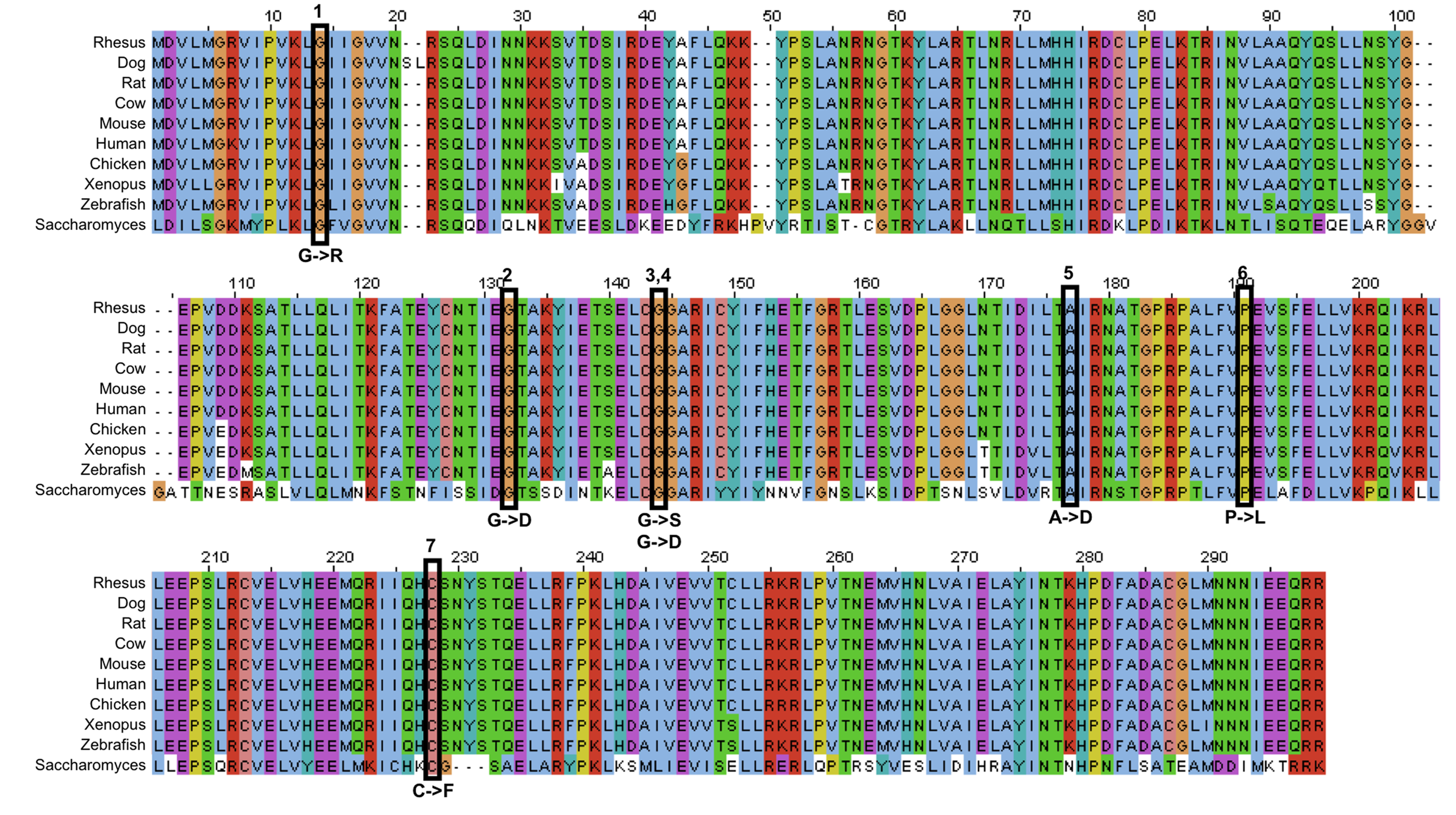

Supplement: Figure S1 — Alignment of domain M in multiple species and mutations found in Dnm1l protein homologues that have an effect on mitochondrial morphology. Identical or functionally very similar amino acids are shown grouped together by colour on the basis of size and hydrophobicity. Reported mutations that result in amino acid substitutions are 1, G269R yeast [14]; 2, G385D yeast [14]; 3, G398S yeast [14]; 4, G369D hamster CHO cell line [16]; 5, A400D human [15]; 6, P444L yeast [14]; 7, C452F mouse (Python). (9.96 MB TIF) [file pgen.1001000.s001.tif]

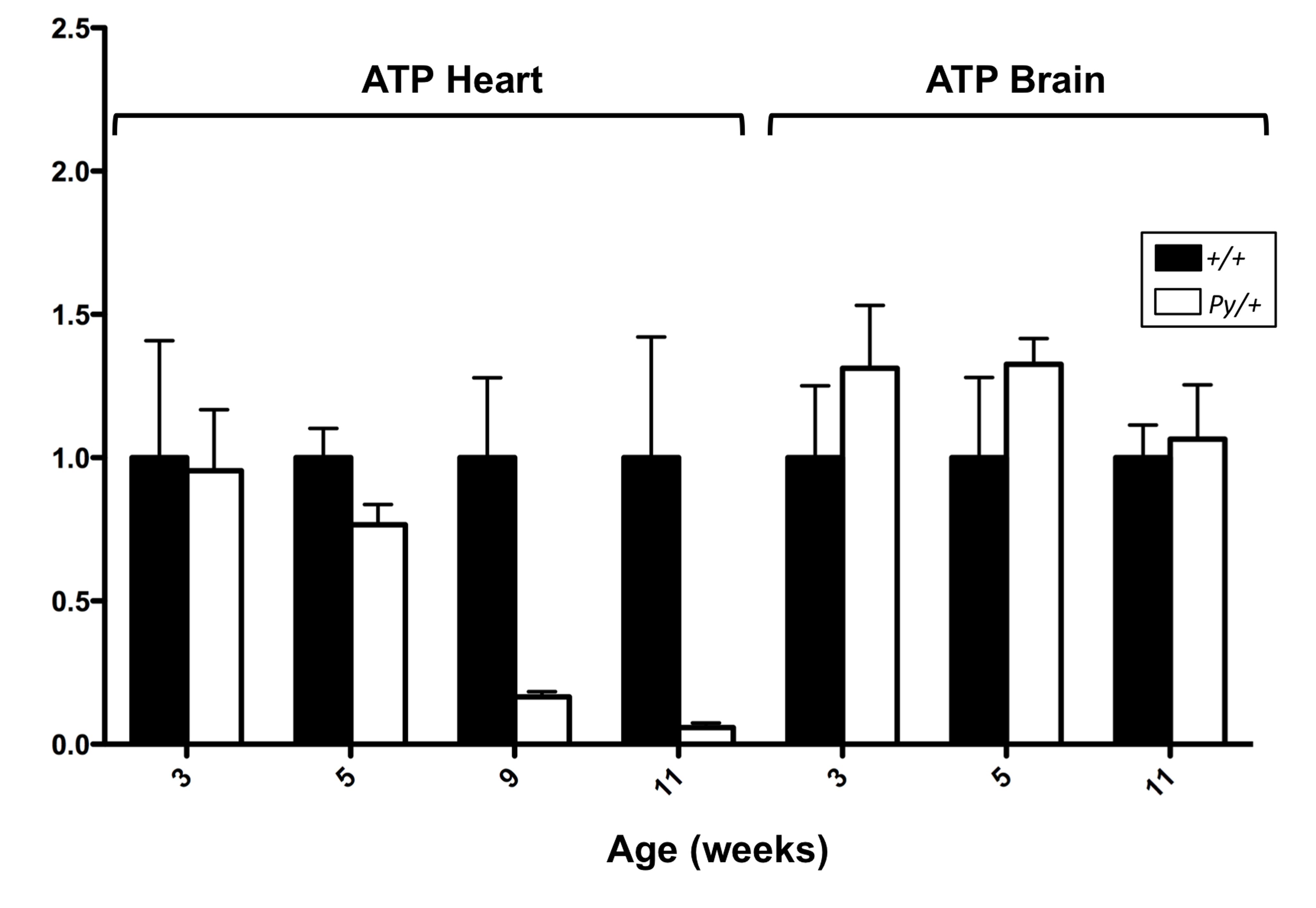

Supplement: Figure S2 — ATP measurements (mean plus/minus SD) in heart and brain samples expressed as level relative to wild type mean, measured using a quantitative bioluminescent method. Tissue samples were minced and then digested for 45 minutes in 10 mM Tris pH 8.0 and 100 µg/ml proteinase K at 55°C. Protein concentrations were estimated using a Bio-Rad DC Protein Assay kit according to the manufacturers instructions. Samples were then normalized based on total protein. ATP was measured using an ATP Luminescence Assay Kit (Invitrogen Corp.) according to the manufacturers instructions. (0.71 MB TIF) [file pgen.1001000.s002.tif]
