# Supplementary material for: A Mutation in the Mitochondrial Fission Gene Dnm1l Leads to Cardiomyopathy
Source: PLoS Genet. 2010 Jun 24;6(6):e1001000. doi: 10.1371/journal.pgen.1001000 (PMC2891719; doi:10.1371/journal.pgen.1001000)
Supplement: Table S1 — Degree of similarity of domain M of mouse dynamin proteins. (0.03 MB DOC) [file pgen.1001000.s003.doc]

**Table S1**

Degree of similarity of domain M of mouse dynamin proteins

|  |  | **Percent Identity** | | | |
| --- | --- | --- | --- | --- | --- |
|  |  | **Dnm1** | **Dnm2** | **Dnm3** | **Dnm1l** |
| **Divergence** | **Dnm1** | - | 37.5 | 35.8 | 34.0 |
|  | **Dnm2** | 121.4 | - | 84.2 | 81.4 |
|  | **Dnm3** | 128.9 | 17.8 | - | 82.5 |
|  | **Dnm1l** | 137.3 | 21.4 | 20.0 | - |
