# Supplementary material for: A Mutation in the Mitochondrial Fission Gene Dnm1l Leads to Cardiomyopathy
Source: PLoS Genet. 2010 Jun 24;6(6):e1001000. doi: 10.1371/journal.pgen.1001000 (PMC2891719; doi:10.1371/journal.pgen.1001000)
Supplement: Table S2 — SHIRPA Assessment of Python mice. (0.04 MB DOC) [file pgen.1001000.s004.doc]

**Table S2**

SHIRPA Assessment of Python mice^1^

|  | *+/+*^2^ | *Py/+*^2^ |
| --- | --- | --- |
|  | (n=12) | (n=12) |
| *Physical Assessment (% abnormal* | | |
| Coat | 0 | 0 |
| Skin | 0 | 0 |
| Head Shape | 0 | 0 |
| Ears | 0 | 0 |
| Vibrissae | 0 | 0 |
| Eyes | 0 | 0 |
| Limbs | 0 | 0 |
| Paws | 0 | 0 |
| Digits | 0 | 0 |
| Nails | 0 | 0 |
| Tail | 0 | 0 |
|  |  |  |
| *Observations and Simple Reflexes (% with)* | | |
| Inactive Body Position | 0 | 0 |
| Tremor | 0 | 0 |
| Defecation | 100 | 92 |
| Transfer Arousal - Freezing Briefly | 67 | 50 |
| Normal Gait | 100 | 100 |
| Tail Horizontal | 100 | 100 |
| Preyer Reflex | 92 | 100 |
| Touch Escape Response | 100 | 100 |
| Normal Grip Strength | 100 | 100 |
| Lack of Positional Passivity | 100 | 100 |
| Trunk Curl | 0 | 0 |
| Pinna Reflex | 100 | 100 |
| Cornea Reflex | 0 | 0 |
| Slow Contact Righting Reflex | 17 | 17 |
| Biting or Aggression | 0 | 0 |
| Vocalisation | 0 | 0 |
| Swimming ability | 100 | 100 |
|  |  |  |
| *Locomotive* activity (mean no. squares + SD) | 3.3+1.5 | 4.2+1.6 |

^1^Based on Ref. 1

^2^Mice were 6-weeks-old at time of analysis

**Reference**

1. Rogers DC, Fisher EM, Brown SD, Peters J, Hunter AJ, Martin JE (1997) Behavioral and functional analysis of mouse phenotype: SHIRPA, a proposed protocol for comprehensive phenotype assessment. Mamm Genome 8: 711-713.
